# Unmet need and intention to use as predictors of adoption of contraception in 10 Performance Monitoring for Action geographies

**DOI:** 10.1016/j.ssmph.2023.101365

**Published:** 2023-02-16

**Authors:** Dana Sarnak, Phil Anglewicz, Saifuddin Ahmed

**Affiliations:** Department of Population, Family and Reproductive Health, Johns Hopkins Bloomberg School of Public Health, USA

**Keywords:** Contraception, Family planning, Demand for contraception, Unmet need for contraception, Panel, Longitudinal, sub-Saharan Africa

## Abstract

The determinants of fertility typically feature demand as the key motivation driver for contraceptive use. Yet relatively little is known about the extent to which demand for contraception predicts future contraceptive use, primarily due to the lack of longitudinal data that captures these measures at different time points. Two ways in which demand is often measured are unmet need and intention to use. Despite its intended use as a population measure, unmet need is commonly used in individual-level analyses and as a marker for individual-level demand for contraception. Few studies have assessed the extent to which unmet need predicts or reflects women's true latent demand as demonstrated by their future contraceptive use; the same is true for intention to use contraception in the future. We expand on previous research to assess whether and the degree to which unmet need and intention to use contraception predict adoption of contraception within a year, among nonusers in ten representative geographies using Performance Monitoring for Action (PMA) data. Findings show that in nine of ten sites, intention to use within a year was significantly associated with subsequent adoption, while in eight of ten sites, unmet need for spacing or limiting was not associated with adoption. Our results are important for programs as they try to identify true dynamic demand for contraception.

## Highlights

• Few studies have assessed women's true latent demand for future contraceptive use.
• In 9/10 geographies, intention to use contraception was associated with adoption.
• In 8/10 geographies, unmet need for contraception was not associated with adoption.
• Intention to use should be incorporated into measures of demand for contraception.

## 1 Introduction

Family planning literature and analytical frameworks of the determinants of fertility typically consider demand as the key motivation driver for contraceptive use (Bongaarts et al., 2012, p. 94; Simmons, 1992). However, relatively little is known about the empirical evidence on the extent to which demand for contraception and unsatisfied demand – measured by unmet need– predict contraceptive use in the future, primarily due to the lack of longitudinal panel data that captures these measures at different time points. However, an understanding of the relationship between demand for contraception and subsequent contraceptive use is important for several reasons. First, this would allow one to identify individuals who eventually fulfill their contraceptive demand and those who do not. The latter group is particularly important, as there may be barriers that inhibit them from contraceptive use that could be addressed through policies and programs. Second, an understanding of this relationship provides insight into the measures of contraceptive demand (Speizer, Bremner, & Farid, 2022), which has emerged as a key development indicator. We focus on two ways in which contraceptive demand is commonly measured (Boydell & Galavotti, 2022): unmet need for contraception and intention to use contraception.

### 1.1 Unmet need

Unmet need for contraception was originally devised as a population-level measure to assess the gap between women's stated desires to delay or cease future childbearing and their nonuse of contraception (Bradley & Casterline, 2014). Despite its intended use as a population-level measure, family planning programmers, researchers and advocates have used unmet need in individual-level analyses as a marker for individual-level demand for contraception (Letamo & Navaneetham, 2015; Senderowicz & Maloney, 2022; Wilopo et al., 2017). For example, numerous studies describe individual-level characteristics and determinants of women with an unmet need for contraception (e.g. Ahinkorah et al., 2020; Mosuse & Gadeyne, 2022).

Unmet need is a complex measure created by family planning researchers and demographers in the 1970s. An underlying assumption of the measure is that nonusers with a desire to delay or limit childbearing are in need of contraception (Sarnak et al., 2020; Speizer, Bremner, & Farid, 2022). Unmet need has been scrutinized and critiqued at length elsewhere, e.g., Bradley & Casterline, 2014; Casterline et al., 1997; Senderowicz & Maloney, 2022. Prominent limitations are that unmet need among nonusers does not include any information from the woman herself on her future plans to adopt contraception, and whether those intentions are in alignment or seemingly in contradiction with her defined or desired “need” for contraception. Others have argued that users, who by definition have no unmet need, may in fact have an unmet need for contraception due to dissatisfaction with their current method (Rominski & Stephenson, 2019; Rothschild et al., 2021). Recent work has also elaborated on the shortcomings of the indicator from rights- and reproductive justice-based frameworks (Senderowicz & Maloney, 2022; Speizer, Bremner, & Farid, 2022); namely that it fails to recognize and respect people's agency and autonomy to freely decide when and whether to have children. While there has been some initial work done to revise the definition of unmet need to incorporate women's stated intention to use contraception in the future (Moreau et al., 2019), there have been calls in the community to develop new measures that take into account women's, men's, and couple's stated family planning needs, including barriers they face and aspects of satisfaction and autonomy (Rominski & Stephenson, 2019; Speizer, Bremner, & Farid, 2022). Further, there are ongoing longitudinal studies aimed to investigate reasons for unmet need, focusing on the shortcomings of current fertility preference measurement and how they may not directly translate to a desire to use contraception (Machiyama et al., 2017).

Despite these critiques, unmet need is still the most widely used metric to measure a current population's demand for contraception, most likely due to its standardization across contexts and continued use as a key family planning progress indicator in global commitments such as the Sustainable Development Goals (SDGs) or FP2030 (United Nations, 2019), as well as its intuitive meaning-although this is often misunderstood (Senderowicz & Maloney, 2022; Speizer, Bremner, & Farid, 2022). However, there are very few assessments of the extent to which unmet need predicts women's true latent demand of contraceptive use (Callahan & Becker, 2014; Curtis & Westoff, 1996; Lutalo et al., 2018; Roy et al., 2003; Sarnak et al., 2020; Speizer, Zavier, et al., 2022), even though some components of unmet need, such as fertility preferences, suggest that it may capture this concept (Chace Dwyer et al., 2022; Srivastava et al., 2019; Tan & Tey, 1994; Tobey et al., 2020). Therefore, this research also informs broader discussions about (1) exactly what unmet need measures, and (2) measuring demand for contraception.

### 1.2 Self-reported intention to use contraception

While unmet need has been revised and reflected upon at length, less attention has been given to studying women's reported intention to use contraception and its relation to subsequent contraceptive use dynamics. In light of a growing body of work on its utility (Babalola et al., 2015; Callahan & Becker, 2014; Moreau et al., 2019; Sarnak et al., 2020), a recent scoping review set out to summarize the state of the evidence on measuring intention to use contraception (Boydell & Galavotti, 2022). The review identified an increasing number of studies using the measure, as well as results linking intention to subsequent contraceptive use. Yet the review also identified the lack of a standard definition or operationalization of the measure. Of note, it highlighted various types and strengths of intentions; specifically, goal intentions, plans people have for some time in the future, versus implementation intentions, ideas people have specific plans for in terms of when, where, and how they will achieve them. Not surprisingly, the review found that studies with survey questions that measured implementation intentions showed positive relationships between intention and use compared with null findings between intention to use in the unspecified future and use.

### 1.3 Existing evidence and gap in literature

The lack of knowledge of the extent to which unmet need and intention to use predict future contraceptive use is primarily due to the lack of longitudinal data. We found only six studies that used longitudinal data to examine how intention to use *and* unmet need (or components of it) influenced subsequent adoption of contraception in low- and middle- income countries (LMICs). Curtis and Westoff (1996) looked at intention to use contraception and subsequent adoption among married women in Morocco over a period of three years. They found that intention to use was a strong predictor of adoption, in addition to the desire to limit births. Roy et al. (2003) used retrospective data to compare behavior with stated childbearing and contraceptive intentions in a baseline and follow up survey in Madhya Pradesh, India, and found that women's intentions about contraceptive use were more definite than their plans about childbearing. Women who intended to use were more likely to use than those who didn't; respondents who intended both not to have children and to use a method were more likely than others to have used a method by the follow up. Using longitudinal data in rural Bangladesh, Callahan and Becker (2014) examined the relationship between intention to use and adoption over a three-year period, among women with and without an unmet need for limiting. They found that regardless of unmet need status, women with an intention to use had increased odds of adoption. Lutalo et al. (2018) looked at the contraceptive use and unintended pregnancy rates of women who had an unmet need at baseline by their intention to use contraception. They found that over half of women with an unmet need and intention to use did not adopt contraception by the next survey round, whom they defined as having an unfulfilled need for contraception. A study using longitudinal data from Uganda showed that nonusers who intended to use contraception adopted faster than those who did not intend, while there was no significant difference in adoption rates between those with and without an unmet need (Sarnak et al., 2020).

Most recently, Speizer, Zavier, et al. (2022) used longitudinal data from married adolescent women in India to examine whether fertility preferences, unmet need status, intention to use and combinations of measures predicted adoption over a period of three years. They found that wanting to space or limit childbearing, having an intention to use, and a combined measure of fertility desire and intention to use family planning were predictive of adoption while unmet need alone was not.

These studies all highlight the importance of intention to use as a marker for adoption, but their conclusions about the utility of unmet need are mixed, and generalizations are further complicated by varying follow up times and selective samples in each study. Additionally, the geographic scope is limited: of these six studies, three were set in Asia, the others used data from two African countries; Morocco and Uganda. Only one study used nationally representative data (Curtis & Westoff, 1996); this is of consequence since most of these studies show that the relationship between demand for contraception and actual use varies by sociodemographic characteristics (like urban/rural residence), which illustrates the value of a diverse sample.

In this study we use geographically representative longitudinal data from ten diverse settings (most of which are in sub-Saharan Africa) to examine the relationship between demand for contraception and future contraceptive use. Our research draws on theoretical frameworks utilized in family planning research which often place intentions as proximal determinants of behavior (Ajzen, 1991; Babalola et al., 2015; Boydell & Galavotti, 2022). Therefore, we expect intention to use contraception to be associated with the subsequent behavior of adoption. The theoretical relationship between unmet need and adoption is less straightforward; given it is based on a mix of future and past preferences for childbearing or wantedness of pregnancies/births, respectively, behaviors such as sexual activity, and perceptions of fecundity, its placement within a conceptual framework modeling behavioral outcomes is uncertain.

This study adds to the growing evidence on the utility of unmet need and intention to use, specifically their value in predicting contraceptive use dynamics. We expand on previous research in two key ways. First, this study examines the intersections between unmet need and intention to use in ten diverse LMIC settings using population-level data, therefore our findings aim to be more generalizable to the region. Second, this study collects data on respondent's intended timing of adoption, thereby adding to the evidence around goal and implementation intentions and subsequent adoption. We hypothesize that unmet need without intention will have a null effect on the probability of adoption, while a woman's intention to use irrespective of her unmet need classification will be associated with an increased probability of adoption.

## 2 Methodology

### 2.1 Data

We used data from the Performance Monitoring for Action (PMA) surveys, which utilize a panel design with embedded cross-sectional surveys to estimate family planning and other health indicators on an annual basis in selected FP2030 country settings. PMA currently operates in eight countries, collecting representative, longitudinal data at the national and/or sub-national level in ten geographies: in Kenya, Burkina Faso, Niger, Uganda, and Côte d’Ivoire; and collects representative data at the sub-national level in India (Rajasthan), Nigeria (Kano and Lagos states only), and Democratic Republic of Congo (Kinshasa and Kongo Central provinces). PMA employs Resident Enumerators (REs), women who live within or nearby the enumeration areas where data are collected, to conduct the surveys. Data are collected on smart phones using the Open Data Kit (ODK) program.

PMA uses a multi-stage stratified cluster design to draw a probability sample of households and females of childbearing age (15–49 years) in all geographies. PMA weights all data to account for study design and non-response, and attrition for the longitudinal data. More information about the PMA study design can be found in Zimmerman et al. (2017) and https://www.pmadata.org/data/survey-methodology.

For this analysis, we use data from the Phase 1 (P1) and Phase 2 (P2) surveys, which were collected between November 2019 and January 2021, with approximately one year between the phases in each geography. The analytical sample in this study includes all women in the P1 and P2 panel. Overall, the PMA surveys have low attrition rates of approximately 20% between the two survey rounds in all geographies (Appendix A1).

Our analytical sample in each country was limited to non-contracepting women at baseline (P1), who were the at-risk population for contraceptive method adoption at the 12- month follow-up (measured at P2). Women who were pregnant or postpartum were included in the sample because both groups of women can be classified as having an unmet need for contraception (e.g. if a women is pregnant with a mistimed or unwanted pregnancy or if a woman is postpartum amenorrheic for up to two years following an unwanted or mistimed birth and not using contraception [Bradley et al., 2012]) and they were also asked about their future intention to use contraception. The percent of pregnant women in the samples ranged from 8.2% in Rajasthan to 14.2% in Uganda.

### 2.2 Measures

Our dependent variable is adoption of contraception. By definition, all women in this study were nonusers of any (modern or traditional) contraception at P1. Therefore, adopters were defined as those women who report using any method of contraception (modern or traditional) at the P2 survey, while those who report nonuse at P2 were defined as continued nonusers. PMA surveys utilize a series of questions to improve the accuracy of contraceptive use reporting and reduce potential underreporting of male or traditional methods. First, like other similar large-scale surveys of family planning (e.g., Demographic and Health Surveys), respondents are primed for the current contraceptive use question with a battery of questions asking if they have heard of each contraceptive method, including probes and images of methods. Respondents are then asked the question: *“Are you or your partner currently doing something or using any method to delay or avoid getting pregnant?”* Additionally, PMA has a follow up question posed to women who respond “no” to the current use question; they are asked a further probe to minimize underreporting of certain methods: *“Just to check, are you or your partner doing any of the following to avoid pregnancy: deliberately avoiding sex on certain days, using a condom, using withdrawal or using emergency contraception?”*

We have two independent variables of interest, all measured at P1: unmet need and intention to use. Like others before us (Sarnak, Tsui, et al., 2021), we conceptualized these measures as concurrent, independent measures in our conceptual models. Our unmet need measure was defined as per the Revised Unmet Need DHS definition (Bradley et al., 2012), and includes the following categories: no unmet need; unmet need for spacing, unmet need for limiting, infecund/menopausal, and non/not-sexually active. In short, women are classified as having an unmet need for spacing or limiting if they are sexually active, fecund women who are not using contraception and want to want to space or limit childbearing, respectively. Women are classified as having no unmet need if they are using contraception (excluded in our study), or if they desire to have children within the next two years. Pregnant and postpartum women are classified on the wantedness status (intended/unintended) of their current or most recent pregnancy/birth. Women who report being infecund, menopausal or not sexually active are considered to have no unmet need. Given the heterogeneity of the group of women with no unmet need, we opted to keep infecund/menopausal and non-sexually active women in separate categories from those with no unmet need based on the desire for a child within two years considering that their demand for contraception and propensity to adopt may not be similar. Further, prior work has criticized the classification of women who self-report infecundity as having no need and quantify the potential error of this assumption (Bradley & Casterline, 2014; Karra, 2022). For most analyses in this study, we separate women with an unmet need for contraception into the specific type (e.g. unmet need for spacing or unmet need for limiting), because we expect women with an unmet need for limiting to be more motivated to avoid pregnancy than those who have an unmet need for spacing; this hypothesis is based on longitudinal research that shows individual preferences for limiting births to be associated with subsequent fertility, while there are less consistent associations between the desire to delay childbearing and subsequent fertility (Cleland et al., 2020).

The intention to use contraception measure was constructed from two questions asked to nonusers. First, all nonusers are asked: *“Do you think you will use a contraceptive method to delay or avoid getting pregnant at any time in the future?*" and response categories are yes; no; don't know; no response. This question is meant to capture, and interviewers are trained to probe about, intention to use any contraceptive method (modern and traditional) in the future. Those who respond “yes” are followed up with a question about timing: *“When do you think you will start using a method?”* to which they can give a month/year response, or a non-numerical response: soon/now; after the birth of this child; don't know; no response. From these questions we created a categorical variable to assess intention to use and timing with the following categories: no intention to use; intention to use within a year (≤ 12 months); intention to use after one year (>12 months).

Finally, to examine whether there was an interaction between unmet need and intention to use, we created a third variable with nine categories that show the cross classification of our two main variables of interest: (1) infecund/menopausal/non sexually active & no intention to use; (2) infecund/menopausal/non sexually active & intention to use >1 year; (3) infecund/menopausal/non sexually active & intention to use ≤ 1 year; (4) no unmet need & no intention to use; (5) no unmet need & intention to use >1 year; (6) no unmet need & intention to use ≤ 1 year; (7) unmet need & no intention to use; (8) unmet need & intention to use >1 year; and (9) unmet need and intention to use ≤ 1 year. Theoretically, one might expect that the combination of demand measures such as unmet need and intention to use may be more important than each individual category (Callahan & Becker, 2014; Curtis & Westoff, 1996). It is only through this cross-classification that we could determine which one is more important, given that our first set of regressions is just the predictive value of one after adjusting for the other. Though existing evidence on the interaction of these two measures is limited, perhaps surprisingly, previous research showed that women with intention to use but no unmet need were more likely to adopt contraception sooner (Sarnak et al., 2020).

In regression models, we adjusted for the following potential confounding covariates that are likely associated with contraceptive use/adoption of contraception: age (15–24 years; 25–34 years; 35+ years), parity (0 children; 1–2 children; 3–4 children; 5+ children), highest schooling level (none/primary; secondary +), household wealth tertile (lowest; middle; highest), rural/urban residence, type of partnership (currently married/living with partner or not married but with a partner/boyfriend; not married, no partner/boyfriend), and previous use of contraception (no/yes).

### 2.3 Analytical approach

First, we compared socio-demographic characteristics of P1 nonusers across sites. Second, we compared the distributions of P2 continued nonusers versus P2 adopters by their P1 unmet need status and their P1 intention to use status, using design-based F-statistics for complex survey analysis to test whether the differences were significant. Third, we used logistic regressions to estimate the odds of P2 adoption versus continued nonuse, by a woman's P1 unmet need status and her intention to use contraception in two different ways. In our first model, we included a woman's unmet need status and her intention to use to examine their independent effects adjusted for confounding covariates. In our second model, we included the cross-classification of these two measures to explore the interaction of these two variables on the odds of adoption. For ease of interpretation, we present predicted probabilities and average marginal effects. The full logistic regression model results (including odds ratios, 95% confidence intervals and p-values) are presented in appendices. In all analyses we used design-based logistic regressions to adjust for higher design-effects (DEFF>1) due to the multi-stage stratified cluster design. We also applied survey weights adjusted for differential loss to follow up using inverse probability weighting methods (Howe et al., 2016).

## 3 Results

### 3.1 Descriptive statistics

As shown in Table 1, most sites were composed of relatively young women, with over 40% of women in all sites except Lagos 15–24 years old, reaching 62% in Rajasthan. Women's highest level of education varied across sites. Over 2/3 of women in Burkina Faso, Côte d’Ivoire, Kano, Niger and Uganda reported their highest education as none or primary, while over 50% of women reported having at least a secondary education in Kinshasa, Kongo Central, Kenya, Lagos and Rajasthan, reaching 93% in Kinshasa. The majority of women lived in rural areas in Burkina Faso, Kenya, Kano, Niger, Rajasthan and Uganda, while the majority lived in urban areas in Côte d’Ivoire.^1^ Types of partnerships ranged by site; over ¾ of women in Burkina Faso, Côte d’Ivoire, Kongo Central, and Kano and Niger reported being currently married, living with their partners or in relationships. Approximately 1/3 or more of women in Kinshasa, Kongo Central, Kenya, Lagos, Rajasthan and Uganda reported not being married nor having a partner/boyfriend. In terms of prior use of contraception, most women reported never having used before. Close to or over ¾ of women in Burkina Faso, Côte d’Ivoire, Kongo Central, Kano, Niger and Rajasthan reported never using before, reaching over 90% of women in Kano and Rajasthan. Just over half of women in Uganda reported prior use (57%).

**Table 1:** Distribution of descriptive characteristics of the sample at Phase 1, across 10 PMA geographies.

Descriptive characteristics at P1		Burkina Faso	Côte d'Ivoire	Kinshasa	Kongo Central	Kenya	Kano	Lagos	Niger	Rajasthan	Uganda
*Individual Characteristics*											
Age	15–24 years	41.8	41.6	50.7	41.2	52.3	43.3	33.4	42.0	62.0	50.7
	25–34 years	28.0	29.3	20.2	25.2	22.4	30.4	32.6	33.3	23.6	25.4
	35+ years	30.2	29.1	29.1	33.6	25.4	26.3	34.0	24.7	14.4	23.8
Highest schooling level	None/Primary	79.2	69.2	7.0	43.1	47.6	67.6	13.3	85.4	33.3	62.5
	Secondary+	20.8	30.8	93.0	56.9	52.4	32.4	86.7	14.6	66.7	37.5
Parity	0 children	28.3	31.1	51.7	29.1	45.7	29.6	43.8	23.2	59.0	38.9
	1-2 children	23.4	30.6	25.2	27.7	24.4	18.2	30.1	22.1	26.6	23.7
	3-4 children	20.3	18.7	14.1	23.0	14.5	15.0	20.9	22.1	11.6	15.0
	5 plus children	28.1	19.6	9.0	20.2	15.3	37.2	5.2	32.6	2.7	22.5
*Household characteristics*											
Household wealth tertile	Lower	36.0	32.4	30.0	36.0	38.7	32.1	35.3	32.7	25.7	34.0
	Middle	33.3	29.6	35.3	33.5	33.6	35.3	30.8	32.8	34.3	32.2
	Highest	30.7	38.0	34.7	30.5	27.6	32.7	33.9	34.5	40.0	33.8
Residence	Rural	79.8	40.0	–	–	72.2	67.6	–	82.4	74.1	73.5
	Urban	20.2	60.0	–	–	27.8	32.4	–	17.6	25.9	26.5
*Partner characteristics*											
Type of partner	Currently married or partner/boyfriend	82.2	78.7	62.6	71.4	59.6	87.9	69.9	88.3	52.7	66.1
	Not married, no partner/boyfriend	17.8	21.3	37.4	28.6	40.4	12.1	30.1	11.7	47.3	33.9
*Prior use of contraception*											
Ever used contraception	No	73.3	74.2	65.0	77.9	65.4	95.0	70.5	84.9	92.9	57.0
	Yes	26.7	25.8	35.0	22.1	34.6	5.0	29.5	15.1	7.1	43.0
Total N		3501	2178	1111	902	3661	882	665	2335	2159	1922

### 3.2 Bivariate associations

Table 2a, Table 2b presents bivariate associations between the two main independent variables of interest, P1 unmet need and intention to use, and P2 adoption at follow up. Overall, 20–30% of women across all sites had adopted by P2, except in Kano (6%) and Niger (11%).

**Table 2a:** Percentage of P2 continued contraceptive non use and adoption by P1 unmet need status and contraceptive intention to use, Burkina Faso, Côte d'Ivoire, Kinshasa, Kongo Central, Kenya.

P1 independent variable		Burkina Faso				Côte d'Ivoire				Kinshasa				Kongo Central				Kenya			
		Column distribution	Row distribution		p value	Column distribution	Row distribution		p value	Column distribution	Row distribution		p value	Column distribution	Row distribution		p value	Column distribution	Row distribution		p value
		Total	P2 continued nonuser	P2 adopter		Total	P2 continued nonuser	P2 adopter		Total	P2 continued nonuser	P2 adopter		Total	P2 continued nonuser	P2 adopter		Total	P2 continued nonuser	P2 adopter	
Unmet need (UN) status	No UN	34.1	72.0	28.0		27.6	79.1	20.9		16.9	71.0	29.0		15.1	61.9	38.1		17.9	52.4	47.6	
	UN for spacing	24.0	75.2	24.8		22.6	69.9	30.1		15.2	50.0	50.0		26.0	66.2	33.8		13.5	53.7	46.3	
	UN for limiting	5.2	77.0	23.0		5.3	69.9	30.1		4.2	73.2	26.8		9.7	61.0	39.0		9.1	52.8	47.2	
	Infecund/menopausal	12.6	93.4	6.6		14.2	89.8	10.2		9.3	93.5	6.5		19.4	84.9	15.1		10.6	78.2	21.8	
	Not sexually active	24.1	86.3	13.7	<0.01	30.3	78.6	21.4	<0.01	54.5	81.3	18.7	<0.01	29.7	85.4	14.6	<0.01	48.9	82.1	17.9	<0.01
Intention to use	No	36.7	89.8	10.2		41.2	87.3	12.7		36.2	86.2	13.8		43.7	79.7	20.3		38.4	81.5	18.5	
	Yes, not within year	31.0	82.6	17.4		29.8	81.4	18.6		41.9	78.9	21.1		26.0	78.9	21.1		38.5	76.6	23.4	
	Yes, within year	32.3	64.1	35.9	<0.01	28.9	59.1	40.9	<0.01	22.0	49.1	50.9	<0.01	30.3	60.3	39.7	<0.01	23.1	39.0	61.0	<0.01
Total	Total		79.3	20.7			77.4	22.6			75.0	25.0			73.6	26.4			69.8	30.2

**Table 2b:** Percentage of P2 continued contraceptive non use and adoption by P1 unmet need status and contraceptive intention to use, Kano, Lagos, Niger, Rajasthan, Uganda.

P1 independent variable		Kano				Lagos				Niger				Rajasthan				Uganda			
		Column distribution	Row distribution		p value	Column distribution	Row distribution		p value	Column distribution	Row distribution		p value	Column distribution	Row distribution		p value	Column distribution	Row distribution		p value
		Total	P2 continued nonuser	P2 adopter		Total	P2 continued nonuser	P2 adopter		Total	P2 continued nonuser	P2 adopter		Total	P2 continued nonuser	P2 adopter		Total	P2 continued nonuser	P2 adopter	
Unmet need (UN) status	No UN	34.5	93.3	6.7		25.7	70.2	29.8		47.6	87.5	12.5		16.4	64.5	35.5		24.4	65.7	34.3	
	UN for spacing	17.3	87.1	12.9		9.5	52.6	47.4		19.2	82.9	17.1		7.4	64.2	35.8		17.4	59.1	40.9	
	UN for limiting	6.2	95.9	4.1		8.1	60.6	39.4		1.9	96.1	3.9		7.8	43.3	56.7		9.1	62.8	37.2	
	Infecund/menopausal	16.0	97.1	2.9		13.3	81.4	18.6		13.4	93.0	7.0		16.4	79.2	20.8		6.8	84.2	15.8	
	Not sexually active	26.1	99.2	0.8	<0.01	43.3	85.7	14.3	<0.01	17.9	99.8	0.2	<0.01	52.0	98.9	1.1	<0.01	42.3	84.6	15.4	<0.01
Intention to use	No	65.8	98.2	1.8		37.8	75.8	24.2		69.1	92.6	7.4		44.3	86.8	13.2		27.0	85.4	14.6	
	Yes, not within year	16.4	87.6	12.4		48.8	78.2	21.8		16.2	84.5	15.5		38.2	89.5	10.5		41.9	78.1	21.9	
	Yes, within year	17.8	83.5	16.5	<0.01	13.4	52.7	47.3	<0.01	14.7	76.7	23.3	<0.01	17.5	48.2	51.8	<0.01	31.1	53.9	46.1	<0.01
Total			93.8	6.2			73.9	26.1			88.9	11.1			81.1	18.9			72.6	27.4	

The distribution of unmet need status at P1 varied by site. Levels of unmet need were highest in Burkina Faso, Côte d’Ivoire, Kongo Central, and Uganda, where over ¼ women had an unmet need, mostly for spacing. Unmet need was lowest in Rajasthan (15%). Seven percent (Uganda) to 19% (Kongo Central) of the sample reported being infecund or menopausal. In Kinshasa, Kenya, and Rajasthan over half the sample was not sexually active.

Approximately 30–40% of nonusers had no intention to use contraception at P1, except in Niger and Kano, where 70% and 65% reported no intention, respectively. Regarding the timing of their intention, most women with an intention to use did not intend to adopt within the year, except for in Burkina Faso, Côte d’Ivoire, Kongo Central and Niger where the percentages who intended to use within the year or after the year were about even.

Unmet need status at P1 was associated with whether a woman continued as a nonuser or adopted across all countries. In Côte d’Ivoire, Kinshasa, Kano, Lagos, Rajasthan and Uganda, higher percentages of women with an unmet need for spacing or limiting adopted than the other categories. In Côte d’Ivoire and Kenya those with no unmet need had similar percentages adopting by P2 as those with an unmet need. Women who reported being infecund/menopausal at P1 were least likely to adopt; these ranged from 7% in Burkina Faso, Kinshasa & Niger to 22% in Kenya. Not many non-sexually active women adopted by P2, though this varied by country; from <1% in Niger to 21% in Côte d’Ivoire.

Intention to use at P1 was associated with adoption in all sites. Across all sites, women who said they intended to adopt within the year were more likely to have adopted by P2 compared to the other two categories. The lowest percentages of women who did not intend to use adopted across all sites.

### 3.3 Multivariate associations

**Fig. 1:**
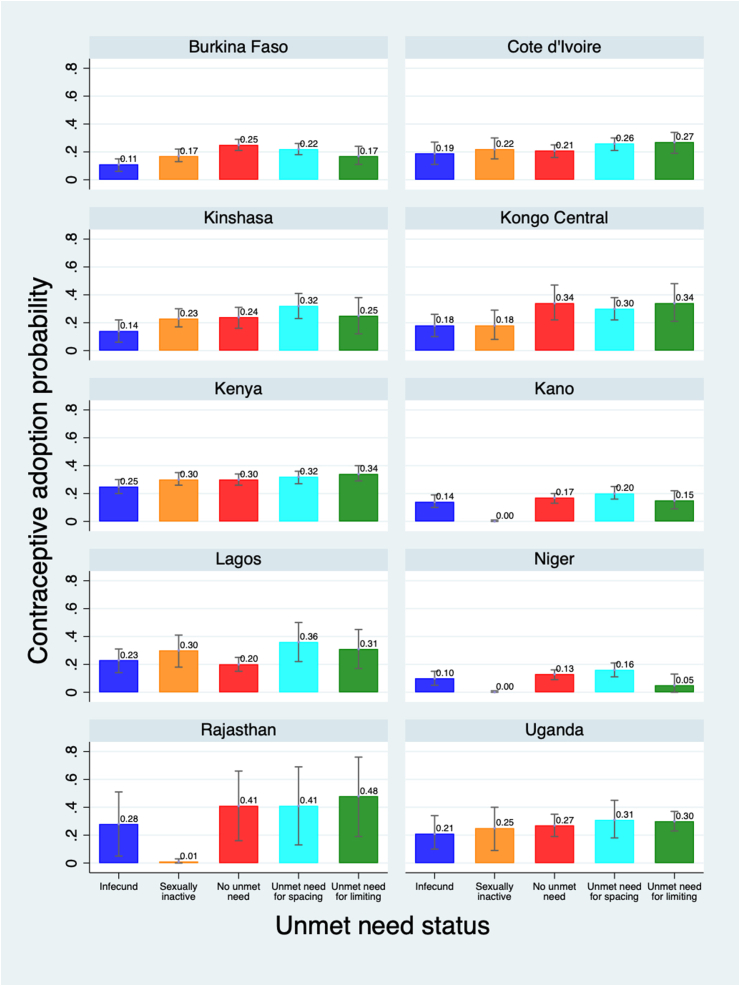
Predicted probability of adoption of contraception at P2, among P1 nonusers, by P1 unmet need status in 10 PMA geographies. Notes: Models are adjusted for women's P1 age, parity, highest schooling level, household wealth tertile, urban/rural residence and prior family planning use.

Fig. 1 and Appendix A2 shows the predicted probability of adoption of contraception by a woman's P1 unmet need status, after adjusting for her age, parity, highest education level, household wealth tertile, urban/rural residence, prior contraception use, and intention to use at baseline. Across almost all sites, there was no relationship between an unmet need for spacing or limiting and probability of adoption. While in eight of ten sites, either those with an unmet need to space or limit had the highest probability of adoption, overlapping 95% confidence intervals show that these differences across groups were not significant. Only in Lagos was having an unmet need for spacing associated with an increased probability of adoption compared to those with no unmet need (36% vs 20%, respectively). In Burkina Faso, having an unmet need for limiting was associated with a reduced probability of adoption compared to those with no unmet need (25% versus 17%). In Burkina Faso and Kongo Central, women who reported being infecund/menopausal had a reduced probability of adoption compared to those with no unmet need (14% and 17% less, respectively). In Burkina Faso, Kano, Niger and Rajasthan, women who reported being not sexually active had a decreased probability of adoption compared to those with no unmet need, ranging from 8% less in Burkina to 40% less in Rajasthan.

**Fig. 2:**
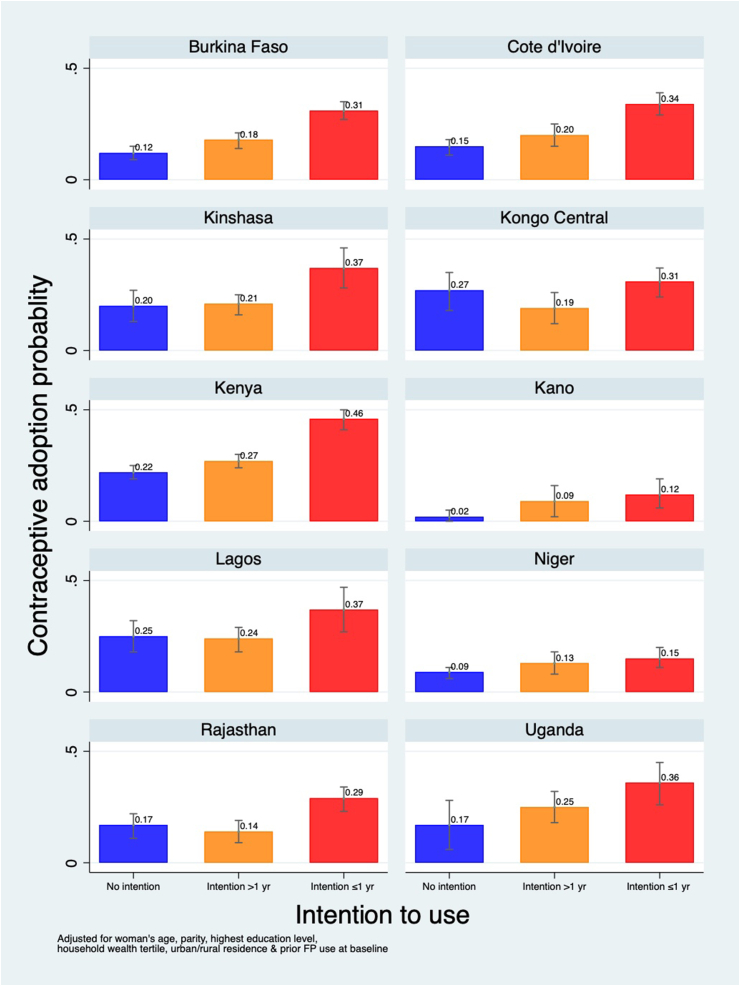
Predicted probability of adoption of contraception at P2, among P1 nonusers, by P1 intention to use in 10 PMA geographies. Notes: Models are adjusted for women's P1 age, parity, highest schooling level, household wealth tertile, urban/rural residence and prior family planning use.

Fig. 2 and Appendix A2 show the predicted probability of adoption of contraception by a woman's P1 intention to use, after adjusting for her age, parity, highest education level, household wealth tertile, urban/rural residence, prior contraception use, and unmet need status at baseline. Across all sites except Kongo Central, women who reported having an intention to use contraception within the year had increased probabilities of adoption in P2 compared to those with no intention to use. Specifically, compared to those with no intention, women with an intention to use within the year had increased probabilities of adoption, e.g., from 9% to 15% in Niger to 22% to 46% in Kenya. In two sites (Burkina Faso and Kenya) having an intention to use, even if it was not within the year, was also associated with an increased probability of adoption compared to no intention to use. Appendix A3 shows the full logistic regression models on which these predicted probabilities are based, and includes odds ratios, 95% confidence intervals and p-values.

**Fig. 3:**
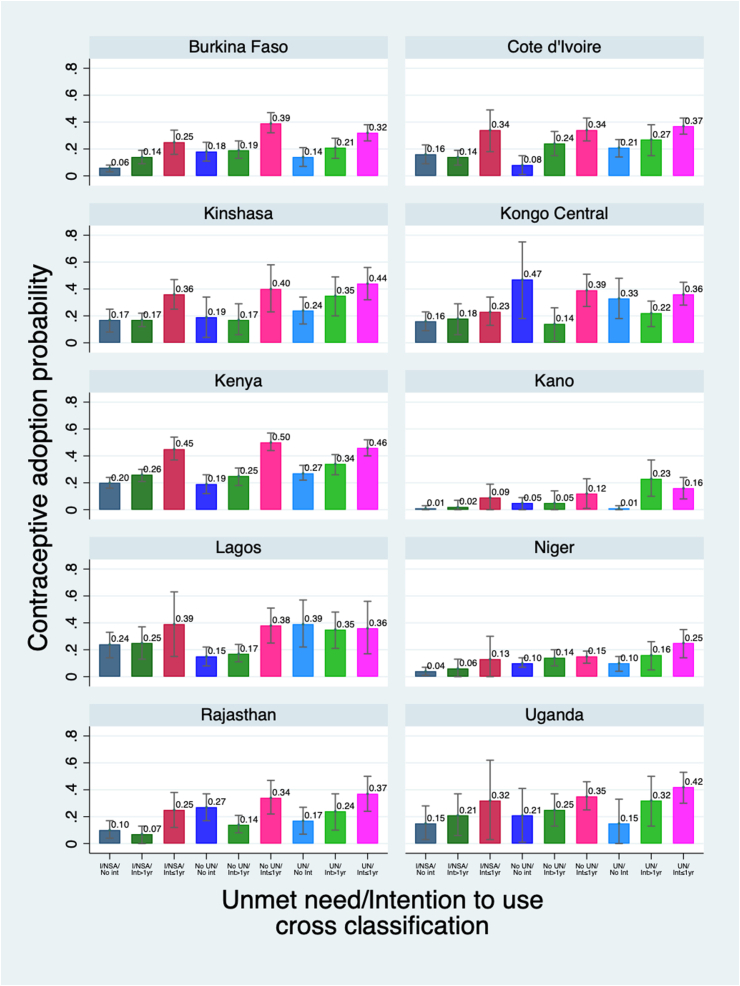
Predicted probability of adoption of contraception at P2, among P1 nonusers, by P1 unmet need-intention to use cross classification in 10 PMA geographies Notes: Models are adjusted for women's P1 age, parity, highest schooling level, household wealth tertile, urban/rural residence and prior family planning use. Sample sizes in Kongo Central are small, particularly in the no unmet need, no intention to use category (n = 41) and produce wide confidence intervals.

Fig. 3 and Appendix A4 show the probability of adoption using the unmet need-intention to use cross-classification, after adjustment for woman's age, parity, highest education level, household wealth tertile, urban/rural residence, & prior contraception use at baseline. In four of ten sites (Burkina Faso, Kenya, Niger, Uganda), regardless of the unmet need status (infecund/not sexually active; no unmet need; and unmet need), there was a clear gradient in the predicted probability of adoption based on whether a woman had an intention to use and the timing of the intention; women with no intention had the lowest probabilities of adoption, while those with an intention to adopt within a year had the highest probability of adoption, across any unmet need category. In another four sites (Côte d’Ivoire, Kano, Kinshasa and Rajasthan), this pattern was mostly seen, with 1–2 exceptions. Only in Lagos, did women with unmet need have the highest probabilities of adoption, regardless of their intention to use. Of note, confidence intervals in all sites were large. Appendix A5 shows the full logistic regression model, odds ratios, 95% confidence intervals and p-values.

## 4 Discussion

Measures of demand for contraception are critical for assessing progress in family planning programs, but there is still widespread debate about how to best capture this concept. This research contributes to the literature on measurement of contraceptive demand in LMICs by testing the extent to which unmet need and intention to use (and the interaction between these measures) predict contraceptive use in the future. To do so, we used longitudinal data from ten geographies.

Our results show substantial differences in predicting future contraceptive use between our two measures of interest. Overall, intention to use is generally more accurate in predicting future contraceptive use than unmet need. We found that in nine of ten sites, intention to use within a year was significantly associated with subsequent adoption. In nearly all sites those with an intention to use had a higher probability of adoption than those without an intention to use. Turning to unmet need, in eight of ten sites, unmet need for spacing or limiting was not associated with adoption in the future. Only in Lagos and Niger was unmet need for spacing associated with greater odds of contraceptive use in the future. There were no statistically significant results for the relationship between unmet need for limiting and future contraceptive use in any of the ten settings. The results of the interaction between the two measures further supported these findings; though confidence intervals were large, those with an intention to use within the year were consistently more likely to adopt regardless of unmet need status in most sites.

Our findings reinforce previous studies that show intention to use to be a strong predictor of subsequent adoption. More specifically, our results show that intention to use within the year is linked to subsequent adoption, supporting behavioral theories around the strength of different types of intentions and the realization of intentions (Gollwitzer, 1999). Our study supports that implementation intentions are particularly strong predictors of adoption, believed to be a result of the type of planning needed to bridge the intention to behavior gap. Of note, these associations persisted across nine geographically and culturally diverse settings with varying levels of contraceptive use and service environments, indicating the salience of this measure as a marker of adoption.

Like prior studies (Sarnak et al., 2020; Speizer, Zavier, et al., 2022) we found that unmet need alone was not a predictor of contraceptive use. While there may be predictive value in some components of the unmet need indicator (measures on fertility intentions, wantedness of pregnancies, and marital status, among others), unmet need itself didn't perform as a predictor of adoption in any site except Lagos. In fact, the interaction revealed that once intention was accounted for, specifically the timing of the intention to use, those categorized with or without unmet need had equal probabilities subsequent adoption. This finding represents a disconnect between the components of unmet need and researcher's assumptions about the latent demand for contraception. For example, many women are classified as having an unmet need if they desire to space or limit childbearing. The assumption that this fertility intention translates to a latent demand for contraception is problematic for a variety of reasons (Fabic, 2022; Sarnak, Tsui, et al., 2021; Speizer, Bremner, & Farid, 2022); adoption of contraception is a multifaceted behavior that may seemingly contradict a woman's fertility intention as it can depend on her assessment of potential side effects of contraception (Diamond-Smith et al., 2012; Schwarz et al., 2019; Zimmerman et al., 2021, 2022); partner factors (Sarnak, Wood, et al., 2021; Staveteig, 2017); exposure to sex (Bell & Bishai, 2017); perceptions of fecundity (Bell & Gemmill, 2021); and the (in)stability of fertility intentions (Aiken et al., 2016; Huber et al., 2017; Müller et al., 2022; Staveteig, 2017). Theoretically, the null relationship between unmet need and adoption also makes sense—unmet need's placement in a conceptual framework or the causal pathway is unclear given that its various components are drawn from past and future preferences, past and future behaviors, and perceptions of fecundity.

Another explanation for unmet need's poor predictive ability could be due to our follow up period of one year; specifically that some women who were categorized at P1 as having no unmet need for contraception (e.g. with a desire to have children sometime in the next **two** years) may have intended to use contraception and subsequently adopted within the **first** year to space childbearing, a shortcoming of the unmet need measure that has also been pointed out elsewhere (e.g. Ross & Winfrey, 2001; Roy et al., 2003). This group of women who are classified as having no unmet need but who have an intention to use, may be missed as a motivated group of users, as pointed out by Callahan and Becker (2014). In the same vein, our finding that close to 1/3 of women who were not sexually active at P1 – women who are often excluded from analyses or categorized as having no unmet need – subsequently adopted in Kenya and Lagos highlights another group of women with a demand for contraception currently overlooked in the unmet need measure.

We acknowledge some limitations in this research. First, we may have missed some contraceptive adoption during the year between surveys, particularly women who may have adopted and discontinued methods. These women would be misclassified as continued nonusers, when in fact they had adopted. The directionality of this bias is unknown. Second, we did not adjust for changes in fertility intentions or intentions to use contraception over the observation period; while we believe this could have accounted for some of the continued nonuse, these analyses were out of the scope of this paper. Third, potential discrepancies in reporting of method use, particularly underreporting of certain methods, including traditional methods, male methods, and/or permanent methods, has been shown to misclassify women's unmet need or contraceptive use status(Staveteig, 2017). Therefore, some women in our study may be similarly misclassified in either survey. However, PMA surveys use various strategies to minimize underreporting of use as outlined above. Fourth, we were unable to account for either local or national level family planning programs that may have influenced whether certain groups of women were differentially targeted for adoption. Finally, most data collection took place during the start of the COVID-19 pandemic; therefore, there may be some unobservable impact of COVID-19. However, limited research on the effects of COVID-19 on contraceptive use patterns show that while health care systems were certainly disrupted by the pandemic and subsequent lockdowns, these disruptions were brief and followed by rapid rebounds and stabilizations within months (Fuseini et al., 2022a, 2022b; Leight et al., 2021; Makumbi et al., 2021; Shuka et al., 2022).

Our results are important for researchers as they try to construct valid measures of the dynamic demand for contraception, as well as for programs and countries in their goals of fulfilling this demand. In terms of research implications, while our results support that a woman's intention to use within the year is a better predictor of contraceptive uptake than unmet need, even women who stated an intention to adopt within a year at P1 had relatively low probabilities of adoption, reaching only a high of 47% in Kenya. Therefore, our study also illuminates the complexity in predicting the demand for contraception, even over a period as short as one year. Future work should investigate why women who intend to use in the short-term do not adopt; whether their continued nonuse is a result of change in time varying measures such as fertility preferences or contraceptive intentions, or whether it could be related to the service or provider environment, building in the work of Roy et al., 2003.

Our research also has practical programmatic implications. First, surveys that measure intention to use contraception should also ask about intended timing of use, given the predictive utility of these implementation intentions. Second, our results are important for family planning programming; women who have a self-identified demand for contraception and subsequently do not adopt would be best suited as intervention targets.

Our research supports the call for researchers, policy makers, and advocates, to reassess the value of unmet need in the family planning field, and to utilize and refine other indicators that reflect patient-centered measures of contraception demand (Fabic, 2022; Speizer, Bremner, & Farid, 2022). Family planning program's reliance on the unmet need measure may be misguided theoretically (Fabic, 2022; Senderowicz & Maloney, 2022; Speizer, Bremner, & Farid, 2022), and our results further indicate its limited utility on future behavior. On the other hand, a woman's reported intention to use represents a measure that is self-identified (Speizer, Bremner, & Farid, 2022), represents autonomous decision-making (Senderowicz & Maloney, 2022), and has a positive association with her propensity to adopt in the future.

## Ethical statement

Performance Monitoring for Action (PMA) received ethical approval from institutional review boards in each country including the Comité d'Ethique Institutionnel Pour La Recherche en Santé (Burkina Faso), École Nationale de Statistiques et d'Economie Appliquee of Abidjan (Côte d'Ivoire), Kenyatta National Hospital-University of Nairobi Ethics Research Committee (Kenya), the Comité d'Ethique Ecole de Sante Publique Universite de Kinshasa (DRC), Makerere School of Public Health and the Uganda National Council for Science and Technology (Uganda), Kano State Ministry of Health (Nigeria-Kano); The Lagos State University Teaching Hospital Heath Research Ethics Committee (Nigeria-Lagos), Ministere de la Sante Publique Comite National d'Ethique pour La Recherce en Sante (CNRS) (Niger), Indian Institute of Health Management Research Institutional Review Board for the Protection of Human Subjects (Rajasthan), and the Johns Hopkins Bloomberg School of Public Health (USA). The authors used publicly available deidentified data for this analysis.

## Funding

This work was supported by 10.13039/100000865Bill & Melinda Gates Foundation grant number OPP1163880. The funding body had no role in the design of the study, data collection, analysis, and interpretation of data and in writing the manuscript.

## Author statement

Dana Sarnak: Conceptualization, Formal Analysis, Writing – Original Draft, methodology, data curation, software. Phil Anglewicz: Supervision, Writing – Review & Editing, Project administration, Fund acquisition. Saifuddin Ahmed: Supervision, Writing – Review & Editing, software.

## Declaration of competing interest

None.

## Data Availability

The datasets analyzed in this study are available in the Performance Monitoring for Action (PMA) repository, https://www.pmadata.org/, and can be obtained for free by requesting access online.

## Footnotes

1 The PMA sample in Kinshasa is urban only. The sample in Kongo Central includes both urban and rural areas, but urban/rural residence is not included in the data for Kongo Central as it is missing from the sampling frame.
