# Supplementary material for: Unmet need and intention to use as predictors of adoption of contraception in 10 Performance Monitoring for Action geographies
Source: SSM Popul Health. 2023 Feb 16;22:101365. doi: 10.1016/j.ssmph.2023.101365 (PMC9996096; doi:10.1016/j.ssmph.2023.101365)
Supplement: Multimedia component 1 [file mmc1.pdf]

APPENDICES

Appendix A1. Distribution of percentages of attrition and socio-demographic characteristics of women in P1 full sample, P2 follow-up sample, and P2 lost to follow up in 10 PMA geographies

		Age (mean)	Education		Parity (mean)	Household wealth tertile			Residence		Marital status		Percent relocated and completed interview	N
			None/ Primary	Secondary+		Lower	Middle	Highest	Urban	Rural	Not married	Married/ living with partner		
BURKINA FASO	P1 Full sample	28.9	77.3	22.7	3.0	34.1	32.4	33.5	23.3	76.7	24.2	75.8	81%	6765
	P2 follow up sample	29.2	78.5	21.5	3.2	34.5	33.3	32.1	21.9	78.1	21.1	78.9		5487
	P2 lost to follow up	27.3	71.0	29.0	2.2	31.9	27.8	40.3	30.4	69.6	39.4	60.6		1278
COTE D'IVOIRE	P1 Full sample	28.7	67.1	32.9	2.2	29.6	31.2	39.3	60.1	39.9	37.4	62.6	73%	4252
	P2 follow up sample	29.0	67.8	32.3	2.4	29.2	31.8	39.0	60.2	39.8	34.3	65.7		3111
	P2 lost to follow up	27.6	65.2	34.7	1.6	30.6	29.4	40.0	59.8	40.2	47.0	53.0		1141
KINSHASA	P1 Full sample	28.3	7.7	92.3	1.7	31.2	33.4	35.4	--	--	57.4	42.6	76%	2634
	P2 follow up sample	28.2	7.3	92.7	1.7	29.9	34.2	35.8	--	--	57.6	42.4		2004
	P2 lost to follow up	28.7	8.9	91.1	1.8	35.4	30.6	33.9	--	--	56.9	43.1		630
KONGO CENTRAL	P1 Full sample	29.3	40.2	59.7	2.1	30.7	32.4	37.0	--	--	36.6	63.4	78%	1967
	P2 follow up sample	29.4	40.3	59.6	2.3	31.1	33.8	35.1	--	--	33.8	66.2		1531
	P2 lost to follow up	28.6	39.9	60.1	1.0	28.9	26.9	44.3	--	--	47.7	52.3		436
KENYA	P1 Full sample	28.8	49.8	50.3	2.4	35.6	34.5	29.9	30.2	69.8	40.9	59.1	73%	9549
	P2 follow up sample	29.4	52.7	47.3	2.6	37.8	25.9	25.9	26.4	73.6	36.6	36.6		7014
	P2 lost to follow up	27.0	41.3	58.7	1.7	29.1	29.6	41.3	41.1	58.9	53.3	46.7		2535
KANO	P1 Full sample	27.8	66.0	34.0	3.7	29.6	33.7	36.7	35.7	64.3	23.9	76.1	89%	1126
	P2 follow up sample	27.9	67.0	33.0	3.8	29.1	34.6	36.3	34.9	65.1	22.5	77.5		1001
	P2 lost to follow up	27.5	57.6	42.5	3.3	34.8	24.6	40.6	42.9	57.1	36.6	63.4		125
LAGOS	P1 Full sample	31.0	11.7	88.1	1.8	33.6	31.8	34.6	--	--	39.4	60.6	75%	1501
	P2 follow up sample	31.4	11.3	88.5	1.9	32.1	32.9	35.0	--	--	34.7	65.3		1130
	P2 lost to follow up	29.7	12.8	87.2	1.5	37.9	28.7	33.5	--	--	52.7	47.3		371
NIGER	P1 Full sample	27.6	84.5	15.5	3.2	31.6	32.0	36.4	19.0	81.0	16.2	83.8	77%	3661
	P2 follow up sample	27.8	85.6	14.4	3.3	32.6	32.3	35.0	18.0	82.0	14.3	85.7		2827
	P2 lost to follow up	26.7	80.2	19.9	2.7	27.5	30.8	41.7	22.9	77.1	23.6	76.4		834
RAJASTHAN	P1 Full sample	29.4	49.7	50.3	1.8	27.0	34.5	38.5	24.9	75.1	25.9	73.5	83%	5464
	P2 follow up sample	29.7	51.9	48.1	1.8	28.3	34.3	37.3	22.2	77.8	23.8	75.6		4541
	P2 lost to follow up	28.2	39.7	60.3	1.4	21.0	35.5	43.5	37.6	62.4	35.6	63.6		923
UGANDA	P1 Full sample	27.6	61.9	38.1	2.7	31.1	33.1	35.9	28.9	71.1	40.9	59.1	76%	3990
	P2 follow up sample	28.5	64.1	35.9	3.0	32.4	34.8	32.9	26.0	74.1	36.8	63.2		3042
	P2 lost to follow up	24.8	55.2	44.8	1.8	27.1	27.6	45.3	38.3	61.7	54.0	46.0		948

Appendix A2. Predicted probabilities for adjusted model 1 (Adjusted predicted probability of adopting contraception, by unmet need and intention to use, among nonusers at P1) in 10 PMA geographies

Predicted probabilities (predictive margins)	Unmet need					Intention to use		
	Infecund/ menopausal	Not sexually active	No unmet need	Unmet need for spacing	Unmet need for limiting	No	Yes, not within year	Yes, within year
Burkina Faso	**0.11 (0.06,0.15)**	**0.17 (0.13,0.22)**	0.25 (0.21,0.29)	0.22 (0.18,0.26)	**0.17 (0.11,0.24)**	0.12(0.09,0.15)	**0.18 (0.14,0.21)**	**0.31 (0.27,0.35)**
Côte d'Ivoire	0.19 (0.11,0.27)	0.22 (0.15,0.30)	0.21 (0.16,0.25)	0.26 (0.21,0.30)	0.27 (0.19,0.34)	0.15 (0.11,0.18)	0.20 (0.15,0.25)	**0.34 (0.29,0.39)**
Kinshasa	0.14 (0.06,0.22)	0.23 (0.17,0.30)	0.24 (0.16,0.31)	0.32 (0.23,0.41)	0.25 (0.12,0.38)	0.20 (0.13,0.27)	0.21 (0.16,0.25)	**0.37 (0.28,0.46)**
Kongo Central	**0.18 (0.10,0.26)**	0.18 (0.08,0.29)	0.34 (0.22,0.47)	0.30 (0.22,0.38)	0.34 (0.21,0.48)	0.27 (0.18,0.35)	0.19 (0.12,0.26)	0.31 (0.24,0.37)
Kenya	0.25 (0.20,0.30)	0.30 (0.26,0.35)	0.30 (0.26,0.34)	0.32 (0.27,0.37)	0.34 (0.29,0.40)	0.22 (0.19,0.25)	**0.27 (0.24,0.30)**	**0.46 (0.41,0.50)**
Kano	0.14 (0.10,0.19)	**0.00 (0.00,0.01)**	0.17 (0.13,0.20)	0.20 (0.16,0.25)	0.15 (0.09,0.22)	0.02 (0.00,0.05)	0.09 (0.02,0.16)	**0.12 (0.06,0.19)**
Lagos	0.23 (0.14,0.31)	0.30 (0.18,0.41)	0.20 (0.15,0.25)	**0.36 (0.22,0.50)**	0.31 (0.17,0.45)	0.25 (0.18,0.32)	0.24 (0.18,0.29)	**0.37 (0.27,0.47)**
Niger	0.10(0.05,0.15)	**0.00(0.00,0.01)**	0.13(0.09,0.16)	0.16(0.11,0.21)	0.05(0.00,0.13)	0.09(0.06,0.11)	0.13(0.08,0.18)	**0.15(0.11,0.20)**
Rajasthan	0.28 (0.05,0.51)	**0.01 (0.00,0.03)**	0.41 (0.16,0.66)	0.41 (0.13,0.69)	0.48 (0.19,0.76)	0.17 (0.11,0.22)	0.14 (0.09,0.19)	**0.29 (0.23,0.34)**
Uganda	0.21 (0.10,0.34)	0.25 (0.09,0.40)	0.27 (0.19,0.35)	0.31 (0.18,0.45)	0.30 (0.23,0.37)	0.17 (0.06,0.28)	0.25 (0.18,0.32)	**0.36 (0.26,0.45)**

dy/dx (average marginal effects)	Unmet need					Intention to use		
	Infecund/ menopausal	Not sexually active	No unmet need	Unmet need for spacing	Unmet need for limiting	No	Yes, not within year	Yes, within year
Burkina Faso	**-0.14 (-0.20,-0.08)**	**-0.08 (-0.14,-0.01)**	--	-0.03 (-0.08,0.02)	**-0.08 (-0.15,-0.01)**	--	**0.05 (0.02,0.09)**	**0.19 (0.14,0.24)**
Côte d'Ivoire	-0.02 (-0.12,0.08)	0.02 (-0.08,0.12)	--	0.05 (-0.01,0.11)	0.06 (-0.03,0.16)	--	0.05 (-0.01,0.11)	**0.19 (0.13,0.25)**
Kinshasa	-0.10 (-0.19,0.01)	-0.01 (-0.10,0.09)	--	0.08 (-0.02,0.19)	0.02 (-0.14,0.17)	--	0.01 (-0.07,0.09)	**0.17 (0.08,0.27)**
Kongo Central	**-0.17 (-0.32,-0.01)**	-0.16 (-0.36,0.04)	--	-0.04 (-0.17,0.08)	-0.01 (-0.15,0.14)	--	-0.08 (-0.19,0.04)	0.04 (-0.07,0.15)
Kenya	-0.05 (-0.11,0.01)	0.01 (-0.06,0.07)	--	0.02 (-0.04,0.07)	0.05 (-0.01,0.10)	--	**0.05 (0.01,0.09)**	**0.24 (0.18,0.29)**
Kano	-0.02 (-0.08,0.03)	**-0.16 (-0.20,-0.13)**	--	0.04 (-0.03,0.10)	-0.01 (-0.09,0.07)	--	0.06 (-0.01,0.14)	**0.10 (0.04,0.16)**
Lagos	0.02 (-0.07,0.12)	0.10 (-0.04,0.23)	--	**0.16 (0.03,0.29)**	0.11 (-0.03,0.24)	--	-0.01 (-0.10,0.08)	**0.12 (0.00,0.24)**
Niger	-0.03(-0.08,0.03)	**-0.12(-0.16,-0.09)**	--	0.03(-0.02,0.09)	-0.08(-0.17,0.02)	--	0.05(-0.01,0.10)	**0.07(0.02,0.12)**
Rajasthan	-0.13 (-0.30,0.05)	**-0.40 (-0.66,-0.14)**	--	0.00 (-0.16,0.16)	0.07 (-0.10,0.23)	--	-0.03 (-0.09,0.03)	**0.12 (0.03,0.21)**
Uganda	-0.05 (-0.20,0.10)	-0.03 (-0.22,0.16)	--	0.04 (-0.12,0.20)	0.03 (-0.06,0.12)	--	0.08 (-0.05,0.21)	**0.18 (0.02,0.34)**

Appendix A3. Logistic regression modeling odds of adopting contraception, among nonusers at P1 in 10 PMA geographies

		Burkina Faso				Côte d'Ivoire				Kinshasa				Kongo central				Kenya			
		aOR	Lower CI	Upper CI	p-value	aOR	Lower CI	Upper CI	p-value	aOR	Lower CI	Upper CI	p-value	aOR	Lower CI	Upper CI	p-value	aOR	Lower CI	Upper CI	p-value
Age, years	15-24 (ref)	--				--				--				--				--			
	25-34	1.10	0.72	1.68	0.67	0.52	0.27	1.00	0.05	0.61	0.36	1.02	0.06	**0.58**	**0.39**	**0.87**	**0.01**	0.79	0.56	1.12	0.18
	35+	1.07	0.63	1.81	0.81	**0.39**	**0.21**	**0.73**	**<0.01**	**0.20**	**0.10**	**0.41**	**<0.01**	**0.32**	**0.17**	**0.61**	**<0.01**	0.71	0.48	1.05	0.09
Highest schooling le	None/Primary (ref)	--				--				--				--				--			
	Secondary+	**2.41**	**1.60**	**3.63**	**<0.01**	1.30	0.76	2.22	0.33	1.11	0.60	2.04	0.73	**1.87**	**1.28**	**2.71**	**<0.01**	1.92	**1.53**	**2.42**	**<0.01**
Parity	0 children (ref)	--				--				--				--				--			
	1-2 children	0.96	0.66	1.41	0.84	**2.21**	**1.18**	**4.16**	**0.01**	1.52	0.97	2.36	0.07	1.18	0.69	2.01	0.54	**1.79**	**1.31**	**2.43**	**<0.01**
	3-4 children	0.91	0.55	1.50	0.70	**2.86**	**1.17**	**6.98**	**0.02**	1.39	0.75	2.58	0.29	1.56	0.77	3.18	0.22	**2.11**	**1.41**	**3.16**	**<0.01**
	5 plus children	0.91	0.46	1.79	0.77	**3.76**	**1.09**	**12.96**	**0.04**	1.92	0.83	4.46	0.12	**2.71**	**1.45**	**5.04**	**<0.01**	1.53	0.98	2.38	0.06
Household wealth	Lower (ref)	--				--				--				--				--			
	Middle	0.78	0.60	1.01	0.06	1.23	0.80	1.88	0.34	0.81	0.46	1.42	0.46	0.84	0.47	1.51	0.56	1.04	0.81	1.34	0.76
	Highest	**0.63**	**0.45**	**0.90**	**0.01**	1.30	0.86	1.98	0.22	0.77	0.46	1.28	0.30	1.40	0.82	2.41	0.21	0.87	0.62	1.23	0.43
Residence	Rural (ref)	--				--				--				--				--			
	Urban	**1.72**	**1.31**	**2.26**	**<0.01**	1.58	0.91	2.75	0.10									1.22	0.89	1.66	0.22
Type of partner	Currently married/living with partner (ref)	--				--				--				--				--			
	Not married, no partner/boyfriend	**0.54**	**0.31**	**0.92**	**0.03**	0.82	0.47	1.42	0.47	**0.47**	**0.27**	**0.84**	**0.01**	0.72	0.40	1.28	0.26	**0.34**	**0.25**	**0.47**	**<0.01**
Ever used FP	No (ref)	--				--				--				--				--			
	Yes	**1.82**	**1.30**	**2.55**	**<0.01**	**1.84**	**1.28**	**2.65**	**<0.01**	1.45	0.87	2.42	0.15	**1.75**	**1.03**	**3.00**	**0.04**	**1.77**	**1.40**	**2.24**	**<0.01**
Unmet need status	No unmet need (ref)	--				--				--				--				--			
	Unmet need for spacing	0.83	0.61	1.14	0.26	1.38	0.93	2.05	0.11	1.65	0.89	3.06	0.11	0.80	0.42	1.52	0.49	1.12	0.81	1.54	0.49
	Unmet need for limiting	0.60	0.35	1.01	0.06	1.48	0.80	2.70	0.21	1.11	0.42	2.91	0.84	0.99	0.49	1.99	0.97	1.31	0.94	1.83	0.11
	Infecund/menopausal	**0.33**	**0.18**	**0.58**	**<0.01**	0.88	0.43	1.81	0.73	0.48	0.22	1.06	0.07	**0.38**	**0.15**	**0.92**	**0.03**	0.73	0.48	1.10	0.13
	Not sexually active	**0.59**	**0.38**	**0.93**	**0.02**	1.12	0.57	2.18	0.75	0.97	0.52	1.81	0.93	0.39	0.12	1.27	0.12	1.04	0.69	1.57	0.86
Intention to use	No (ref)	--				--				--				--				--			
	Yes, not within year	**1.58**	**1.16**	**2.15**	**<0.01**	1.47	0.93	2.32	0.10	1.05	0.62	1.78	0.86	0.61	0.29	1.28	0.19	**1.37**	**1.07**	**1.75**	**0.01**
	Yes, within year	**3.56**	**2.61**	**4.86**	**<0.01**	**3.20**	**2.19**	**4.67**	**<0.01**	**2.70**	**1.60**	**4.56**	**<0.01**	1.25	0.68	2.29	0.47	**3.48**	**2.65**	**4.57**	**<0.01**
N		3501				2178				1111				902				3661			

		Kano				Lagos				Niger				Rajasthan				Uganda			
		aOR	Lower CI	Upper CI	p-value	aOR	Lower CI	Upper CI	p-value	aOR	Lower CI	Upper CI	p-value	aOR	Lower CI	Upper CI	p-value	aOR	Lower CI	Upper CI	p-value
Age, years	15-24 (ref)	--				--				--				--				--			
	25-34	0.71	0.25	2.02	0.50	1.03	0.47	2.23	0.94	0.94	0.50	1.78	0.86	0.74	0.46	1.20	0.22	0.67	0.33	1.36	0.25
	35+	0.39	0.13	1.23	0.10	0.55	0.21	1.44	0.21	0.55	0.29	1.07	0.08	0.88	0.38	2.02	0.76	0.50	0.19	1.36	0.16
Highest schooling le	None/Primary (ref)	--				--				--				--				--			
	Secondary+	1.02	0.31	3.35	0.97	0.98	0.39	2.45	0.97	**1.82**	**1.12**	**2.98**	**0.02**	1.76	0.95	3.28	0.07	1.57	0.89	2.75	0.11
Parity	0 children (ref)	--				--				--				--				--			
	1-2 children	2.00	0.27	15.03	0.48	**2.36**	**1.05**	**5.34**	**0.04**	**0.40**	**0.16**	**1.00**	**0.05**	**2.03**	**1.19**	**3.47**	**0.01**	1.83	0.64	5.25	0.24
	3-4 children	1.78	0.25	12.74	0.55	2.28	0.92	5.62	0.07	0.46	0.19	1.09	0.08	**3.21**	**1.46**	**7.09**	**<0.01**	1.94	0.55	6.86	0.28
	5 plus children	2.17	0.31	15.06	0.42	1.57	0.31	7.90	0.58	0.97	0.37	2.58	0.96	2.00	**0.77**	**5.18**	0.15	2.54	0.75	8.57	0.12
Household wealth	Lower (ref)	--				--				--				--				--			
	Middle	**3.90**	**1.14**	**13.36**	**0.03**	**2.72**	**1.58**	**4.68**	**<0.01**	**1.14**	**0.65**	**2.00**	**0.64**	0.81	0.40	1.67	0.57	1.27	0.69	2.33	0.42
	Highest	**4.21**	**1.08**	**16.47**	**0.04**	**2.45**	**1.43**	**4.19**	**<0.01**	**1.87**	**1.06**	**3.33**	**0.03**	1.09	0.61	1.95	0.76	**1.43**	**1.01**	**2.02**	**0.04**
Residence	Rural (ref)	--				--				--				--				--			
	Urban	1.77	0.59	5.30	0.29					1.17	0.67	2.04	0.59	1.71	0.96	3.06	0.07	1.20	0.58	2.47	0.60
Type of partner	Currently married/living with partner (ref)	--				--				--				--				--			
	Not married, no partner/boyfriend	**#######**	**#######**	**#######**	**<0.01**	**0.24**	**0.11**	**0.55**	**<0.01**	**0.30**	**0.03**	**2.76**	**0.28**	2.81	0.29	27.62	0.37	0.52	0.20	1.39	0.18
Ever used FP	No (ref)	--				--				--				--				--			
	Yes	**2.26**	**1.11**	**4.57**	**0.03**	**1.99**	**1.14**	**3.45**	**0.02**	**2.33**	**1.42**	**3.84**	**<0.01**	1.73	0.88	3.38	0.11	1.64	0.95	2.83	0.07
Unmet need status	No unmet need (ref)	--				--				--				--				--			
	Unmet need for spacing	1.80	0.64	5.06	0.25	**2.66**	**1.32**	**5.35**	**0.01**	**1.37**	**0.86**	**2.16**	**0.18**	1.01	0.49	2.05	0.99	1.24	0.52	2.92	0.61
	Unmet need for limiting	0.77	0.12	4.96	0.77	1.99	0.94	4.25	0.07	0.32	0.04	2.52	0.27	1.34	0.64	2.79	0.43	1.17	0.71	1.92	0.51
	Infecund/menopausal	0.58	0.14	2.30	0.42	1.18	0.61	2.26	0.62	0.74	0.39	1.41	0.36	0.54	0.23	1.28	0.16	0.72	0.28	1.84	0.47
	Not sexually active	**0.00**	**0.00**	**0.00**	**0.00**	1.84	0.76	4.46	0.17	**0.02**	**0.01**	**0.08**	**<0.01**	**0.01**	**0.00**	**0.14**	**<0.01**	0.85	0.28	2.59	0.76
Intention to use	No (ref)	--				--				--				--				--			
	Yes, not within year	**4.27**	**1.08**	**16.98**	**0.04**	0.94	0.53	1.66	0.83	1.70	0.91	3.17	0.10	0.76	0.42	1.39	0.38	1.68	0.67	4.18	0.25
	Yes, within year	**6.54**	**2.50**	**17.10**	**<0.01**	**1.97**	**1.01**	**3.83**	**0.05**	**2.06**	**1.26**	**3.36**	**<0.01**	**2.60**	**1.30**	**5.21**	**0.01**	**2.89**	**1.13**	**7.38**	**0.03**
N		882				665				2335				2159				1922			

Appendix A4. Predicted probabilities for adjusted model 2 (Adjusted predicted probability of adopting contraception, by unmet need and intention to use cross-classification, among nonusers at P1) in 10 PMA geographies

	Unmet need/Intention to use cross-classification								
Predicted probabilities (predictive margins)	Infecund/Menopausal/not sexually active & no intention to use	Infecund/Menopausal/not sexually active & intention to use >1 year	Infecund/Menopausal/not sexually active & intention to use ≤1 year	No unmet need & no intention to use	No unmet need & intention to use>1 year	No unmet need & intention to use ≤ 1 year	Unmet need & no intention to use	Unmet need & intention to use >1 year	Unmet need & intention to use ≤ 1 year
Burkina Faso	0.06 (0.03, 0.08)	**0.14 (0.09, 0.19)**	**0.25 (0.16, 0.34)**	**0.18 (0.11, 0.25)**	**0.19 (0.13, 0.26)**	**0.39 (0.32, 0.47)**	**0.14 (0.07, 0.21)**	**0.21 (0.13, 0.28)**	**0.32 (0.26, 0.38)**
Côte d'Ivoire	0.16 (0.09, 0.23)	0.14 (0.08, 0.19)	**0.34 (0.18, 0.49)**	0.08 (0.01, 0.15)	**0.24 (0.15, 0.33)**	**0.34 (0.26, 0.43)**	0.21 (0.14, 0.27)	0.27 (0.15, 0.38)	**0.37 (0.31, 0.43)**
Kinshasa	0.17 (0.08, 0.25)	0.17 (0.12, 0.22)	**0.36 (0.25, 0.47)**	0.19 (0.04, 0.34)	0.17 (0.06, 0.29)	**0.40 (0.23, 0.58)**	0.24 (0.14, 0.34)	**0.35 (0.20, 0.49)**	**0.44 (0.32, 0.56)**
Kongo Central	0.16 (0.09, 0.23)	0.18 (0.06, 0.29)	0.23 (0.13, 0.34)	**0.47 (0.18, 0.75)**	0.14 (0.01, 0.26)	**0.39 (0.27, 0.51)**	**0.33 (0.18, 0.48)**	0.22 (0.12, 0.31)	**0.36 (0.28, 0.45)**
Kenya	0.20 (0.16, 0.24)	**0.26 (0.21, 0.30)**	**0.45 (0.37, 0.54)**	0.19 (0.12, 0.26)	0.25 (0.18, 0.31)	**0.50 (0.44, 0.57)**	**0.27 (0.22, 0.33)**	**0.34 (0.26, 0.41)**	**0.46 (0.40, 0.52)**
Kano	0.01 (0.00, 0.03)	0.02 (0.00, 0.07)	0.09 (0.00, 0.19)	0.05 (0.00, 0.09)	0.05 (0.00, 0.14)	0.12 (0.01, 0.23)	0.01 (0.00, 0.03)	**0.23 (0.10, 0.37)**	**0.16 (0.08, 0.24)**
Lagos	0.24 (0.14, 0.33)	0.25 (0.13, 0.37)	0.39 (0.15, 0.63)	0.15 (0.08, 0.22)	0.17 (0.11, 0.24)	0.38 (0.25, 0.51)	0.39 (0.22, 0.57)	0.35 (0.21, 0.48)	0.36 (0.17, 0.56)
Niger	0.04 (0.01, 0.07)	0.06 (0.00, 0.13)	0.13 (0.00, 0.30)	**0.10 (0.07, 0.14)**	**0.14 (0.08, 0.20)**	**0.15 (0.10, 0.19)**	0.10 (0.04, 0.15)	0.16 (0.05, 0.26)	**0.25 (0.14, 0.35)**
Rajasthan	0.10 (0.04, 0.17)	0.07 (0.00, 0.13)	**0.25 (0.12, 0.38)**	**0.27 (0.17, 0.37)**	0.14 (0.08, 0.21)	**0.34 (0.22, 0.47)**	0.17 (0.07, 0.27)	**0.24 (0.10, 0.37)**	**0.37 (0.24, 0.50)**
Uganda	0.15 (0.03, 0.28)	0.21 (0.06, 0.37)	0.32 (0.03, 0.62)	0.21 (0.01, 0.41)	0.25 (0.13, 0.37)	**0.35 (0.25, 0.46)**	0.15 (-0.02, 0.33)	0.32 (0.13, 0.50)	**0.42 (0.30, 0.53)**
dy/dx (average marginal effects)	Infecund/Menopausal/not sexually active & no intention to use	Infecund/Menopausal/not sexually active & intention to use >1 year	Infecund/Menopausal/not sexually active & intention to use ≤1 year	No unmet need & no intention to use	No unmet need & intention to use>1 year	No unmet need & intention to use ≤ 1 year	Unmet need & no intention to use	Unmet need & intention to use >1 year	Unmet need & intention to use ≤ 1 year
Burkina Faso	--	**0.09 (0.03, 0.14)**	**0.19 (0.10, 0.29)**	**0.12 (0.06, 0.19)**	**0.14 (0.07, 0.20)**	**0.34 (0.25, 0.42)**	**0.09 (0.01, 0.16)**	**0.15 (0.07, 0.23)**	**0.26 (0.20, 0.33)**
Côte d'Ivoire	--	-0.02 (-0.11, 0.07)	**0.18 (0.04, 0.32)**	-0.08 (-0.19, 0.04)	**0.08 (0.01, 0.15)**	**0.19 (0.05, 0.32)**	0.05 (-0.05, 0.15)	0.11 (-0.00,0.22)	**0.21 (0.13, 0.30)**
Kinshasa	--	0.00 (-0.09, 0.09)	**0.19 (0.06, 0.32)**	0.02 (-0.13, 0.17)	0.00 (-0.14, 0.15)	**0.24 (0.04, 0.43)**	0.07 (-0.06, 0.20)	**0.18 (9.01, 0.34)**	**0.27 (0.14, 0.41)**
Kongo Central	--	0.02 (-0.11, 0.15)	0.08 (-0.05, 0.20)	**0.31 (0.00, 0.61)**	-0.02 (-0.18, 0.14)	**0.23 (0.09, 0.38)**	**0.17 (0.02, 0.33)**	0.06 (-0.06, 0.18)	**0.21 (0.09, 0.32)**
Kenya	--	**0.06 (0.01, 0.11)**	**0.26 (0.16, 0.35)**	-0.01 (-0.09, 0.07)	0.05 (-0.03, 0.13)	**0.30 (0.22, 0.38)**	**0.08 (0.01, 0.14)**	**0.14 (0.06, 0.22)**	**0.26 (0.19, 0.33)**
Kano	--	0.02 (-0.03,0.07)	0.08 (-0.01, 0.17)	0.04 (-0.01, 0.09)	0.05 (-0.04, 0.14)	0.11 (-0.01, 0.23)	0.00 (-0.03, 0.03)	**0.23 (0.10, 0.35)**	**0.15 (0.07, 0.23)**
Lagos	--	0.01 (-0.16, 0.19)	0.15 (-0.13, 0.43)	-0.08 (-0.20, 0.03)	-0.06 (-0.18, 0.05)	0.14 (-0.01, 0.29)	0.16 (-0.04, 0.35)	0.11 (-0.06, 0.28)	0.13 (-0.09, 0.34)
Niger	--	0.02 (-0.05, 0.09)	0.09 (-0.08, 0.26)	**0.06 (0.02, 0.11)**	**0.10 (0.03, 0.18)**	**0.11 (0.05, 0.16)**	0.06 (-0.00, 0.12)	0.12 (-0.00, 0.24)	**0.21 (0.09, 0.32)**
Rajasthan	--	-0.04 (-0.11, 0.04)	**0.15 (0.02, 0.28)**	**0.17 (0.05, 0.29)**	0.04 (-0.07, 0.16)	**0.24 (0.07, 0.41)**	0.07 (-0.04, 0.19)	**0.13 (0.00, 0.26)**	**0.27 (0.10, 0.43)**
Uganda	**--**	0.06 (-0.12, 0.24)	0.17 (-0.12, 0.45)	0.05 (-0.16, 0.27)	0.10 (-0.07, 0.27)	**0.20 (0.03, 0.37)**	0.00 (-0.18, 0.18)	0.16 (-0.09, 0.42)	**0.26 (0.08, 0.44)**

Appendix A5. Logistic regression modeling odds of adopting contraception, among nonusers at P1 in 10 PMA geographies

		Burkina Faso				Côte d'Ivoire			
		aOR	Lower CI	Upper CI	pvalue	aOR	Lower CI	Upper CI	pvalue
Age, years	15-24 (ref)	--				--			
	25-34	1.10	0.72	1.68	0.67	**0.50**	**0.25**	**1.00**	**0.05**
	35+	1.00	0.58	1.72	1.00	**0.36**	**0.20**	**0.67**	**<0.01**
Highest Schooling level	None/Primary (ref)	--				--			
	Secondary+	2.42	**1.59**	**3.70**	**<0.01**	1.41	0.89	2.24	0.14
Parity	0 children (ref)	--				--			
	1-2 children	0.92	0.63	1.34	0.65	**2.05**	**1.19**	**3.54**	**0.01**
	3-4 children	0.88	0.53	1.44	0.60	**2.74**	**1.15**	**6.53**	**0.02**
	5 plus children	0.85	0.43	1.68	0.63	**3.57**	**1.12**	**11.39**	**0.03**
Household wealth	Lower (ref)	--				--			
	Middle	0.78	0.61	1.02	0.06	1.24	0.80	1.93	0.33
	Highest	**0.62**	**0.44**	**0.89**	**0.01**	1.38	0.90	2.12	0.14
Residence	Rural (ref)	--				--			
	Urban	**1.76**	**1.33**	**2.32**	**<0.01**	1.56	0.92	2.67	0.10
Type of partner	Currently married/living with partner (ref)	--				--			
	Not married, no partner/boyfriend	0.64	0.37	1.09	0.10	0.96	0.59	1.56	0.87
Ever used FP	No (ref)	--				--			
	Yes	**1.83**	**1.30**	**2.57**	**<0.01**	**1.95**	**1.32**	**2.87**	**<0.01**
Unmet need / Intention to use	Infecund/Menopausal/not sexually active & no intention to use (ref)	--				--			
	Infecund/Menopausal/not sexually active & intention to use >1 yr	**2.91**	**1.65**	**5.13**	**<0.01**	0.82	0.39	1.76	0.61
	Infecund/Menopausal/not sexually active & intention to use <1 yr	**6.07**	**3.15**	**11.71**	**<0.01**	**2.90**	**1.43**	**5.88**	**<0.01**
	No unmet need & no intention to use	**3.90**	**2.15**	**7.07**	**<0.01**	0.46	0.13	1.62	0.22
	No unmet need & intention to use > 1 yr	**4.24**	**2.45**	**7.33**	**<0.01**	**1.75**	**1.11**	**2.75**	**0.02**
	No unmet need & intention to use ≤ 1 yr	**12.17**	**6.72**	**22.04**	**<0.01**	**2.99**	**1.36**	**6.54**	**0.01**
	Unmet need & no intention to use	**2.89**	**1.32**	**6.34**	**0.01**	1.42	0.70	2.88	0.34
	Unmet need & intention to use > 1 yr	**4.66**	**2.24**	**9.69**	**<0.01**	**2.01**	**1.04**	**3.90**	**0.04**
	Unmet need & intention to use ≤ 1 yr	**8.72**	**5.14**	**14.81**	**<0.01**	**3.37**	**1.96**	**5.79**	**<0.01**
N		3501				2178			

		Kinshasa				Kongo Central			
		aOR	Lower CI	Upper CI	pvalue	aOR	Lower CI	Upper CI	pvalue
Age, years	15-24 (ref)	--				--			
	25-34	0.59	0.35	1.01	0.06	**0.58**	**0.38**	**0.89**	**0.01**
	35+	**0.18**	**0.09**	**0.33**	**<0.01**	**0.33**	**0.18**	**0.60**	**<0.01**
Highest Schooling level	None/Primary (ref)	--				--			
	Secondary+	1.08	0.59	1.99	0.80	**1.87**	**1.28**	**2.71**	**<0.01**
Parity	0 children (ref)	--				--			
	1-2 children	1.42	0.90	2.25	0.13	1.25	0.73	2.13	0.41
	3-4 children	1.27	0.67	2.39	0.45	1.78	0.88	3.59	0.11
	5 plus children	1.77	0.74	4.26	0.20	**2.95**	**1.57**	**5.57**	**<0.01**
Household wealth	Lower (ref)	--				--			
	Middle	0.82	0.47	1.44	0.49	0.82	0.45	1.50	0.52
	Highest	0.77	0.46	1.29	0.31	1.37	0.78	2.38	0.27
Residence	Rural (ref)	--				--			
	Urban								
Type of partner	Currently married/living with partner (ref)	--				--			
	Not married, no partner/boyfriend	**0.52**	**0.29**	**0.92**	**0.03**	0.70	0.43	1.14	0.15
Ever used FP	No (ref)	--				--			
	Yes	1.47	0.88	2.45	0.14	**1.81**	**1.02**	**3.19**	**0.04**
Unmet need / Intention to use	Infecund/Menopausal/not sexually active & no intention to use (ref)	--				--			
	Infecund/Menopausal/not sexually active & intention to use >1 yr	1.02	0.52	1.99	0.95	1.17	0.44	3.09	0.75
	Infecund/Menopausal/not sexually active & intention to use <1 yr	**3.03**	**1.41**	**6.52**	**0.01**	1.70	0.75	3.84	0.20
	No unmet need & no intention to use	1.15	0.39	3.38	0.80	**5.27**	**1.18**	**23.52**	**0.03**
	No unmet need & intention to use > 1 yr	1.03	0.35	3.06	0.95	0.84	0.21	3.34	0.81
	No unmet need & intention to use ≤ 1 yr	**3.72**	**1.32**	**10.47**	**0.01**	**3.76**	**1.66**	**8.49**	**<0.01**
	Unmet need & no intention to use	1.62	0.69	3.83	0.26	**2.85**	**1.21**	**6.71**	**0.02**
	Unmet need & intention to use > 1 yr	**2.81**	**1.07**	**7.39**	**0.04**	1.53	0.68	3.46	0.30
	Unmet need & intention to use ≤ 1 yr	**4.43**	**2.10**	**9.38**	**<0.01**	**3.30**	**1.60**	**6.83**	**<0.01**
N		1111				902			

		Kenya				Kano			
		aOR	Lower CI	Upper CI	pvalue	aOR	Lower CI	Upper CI	pvalue
Age, years	15-24 (ref)	--				--			
	25-34	0.76	0.54	1.08	0.12	0.77	0.29	2.06	0.59
	35+	0.69	0.47	1.01	0.06	0.35	0.10	1.22	0.10
Highest Schooling level	None/Primary (ref)	--				--			
	Secondary+	1.92	**1.53**	**2.41**	**<0.01**	1.04	0.32	3.41	0.94
Parity	0 children (ref)	--				--			
	1-2 children	**1.72**	**1.28**	**2.31**	**<0.01**	3.92	0.72	21.34	0.11
	3-4 children	**2.01**	**1.35**	**3.01**	**<0.01**	3.71	0.68	20.40	0.12
	5 plus children	1.49	0.97	2.31	0.07	**4.90**	**1.04**	**22.99**	**0.04**
Household wealth	Lower (ref)	--				--			
	Middle	1.05	0.82	1.35	0.68	**3.92**	**1.18**	**13.09**	**0.03**
	Highest	0.88	0.63	1.24	0.48	**4.71**	**1.18**	**18.80**	**0.03**
Residence	Rural (ref)	--				--			
	Urban	1.22	0.90	1.65	0.21	1.54	0.50	4.68	0.43
Type of partner	Currently married/living with partner (ref)	--				--			
	Not married, no partner/boyfriend	**0.38**	**0.29**	**0.49**	**<0.01**	1.62	0.25	10.39	0.60
Ever used FP	No (ref)	--				--			
	Yes	**1.82**	**1.44**	**2.30**	**<0.01**	**2.95**	**1.32**	**6.59**	**0.01**
Unmet need / Intention to use	Infecund/Menopausal/not sexually active & no intention to use (ref)	--				--			
	Infecund/Menopausal/not sexually active & intention to use >1 yr	**1.47**	**1.06**	**2.03**	**0.02**	3.19	0.17	61.57	0.43
	Infecund/Menopausal/not sexually active & intention to use <1 yr	**3.93**	**2.48**	**6.21**	**<0.01**	**13.99**	**1.59**	**122.80**	**0.02**
	No unmet need & no intention to use	0.95	0.54	1.67	0.86	6.57	0.47	91.43	0.15
	No unmet need & intention to use > 1 yr	1.37	0.83	2.25	0.21	7.83	0.32	194.38	0.20
	No unmet need & intention to use ≤ 1 yr	**4.89**	**3.28**	**7.28**	**<0.01**	19.66	1.00	387.03	0.05
	Unmet need & no intention to use	**1.62**	**1.11**	**2.35**	**0.01**	1.39	0.04	43.26	0.84
	Unmet need & intention to use > 1 yr	**2.25**	**1.45**	**3.49**	**<0.01**	**49.63**	**6.42**	**383.77**	**<0.01**
	Unmet need & intention to use ≤ 1 yr	**4.02**	**2.77**	**5.82**	**<0.01**	28.72	2.23	370.29	0.01
N		3661				882			

		Lagos				Niger			
		aOR	Lower CI	Upper CI	pvalue	aOR	Lower CI	Upper CI	pvalue
Age, years	15-24 (ref)	--				--			
	25-34	0.95	0.44	2.08	0.90	1.01	0.53	1.94	0.97
	35+	0.44	0.18	1.08	0.07	0.61	0.32	1.16	0.13
Highest Schooling level	None/Primary (ref)	--				--			
	Secondary+	0.99	0.38	2.58	0.98	1.54	0.90	2.64	0.11
Parity	0 children (ref)	--				--			
	1-2 children	**2.27**	**1.02**	**5.02**	**0.04**	0.63	0.23	1.73	0.36
	3-4 children	2.05	0.79	5.32	0.14	0.67	0.24	1.87	0.44
	5 plus children	1.40	0.26	7.47	0.69	1.35	0.43	4.26	0.60
Household wealth	Lower (ref)	--				--			
	Middle	**2.65**	**1.54**	**4.57**	**<0.01**	1.21	0.70	2.08	0.50
	Highest	**2.53**	**1.46**	**4.37**	**<0.01**	**2.02**	**1.13**	**3.60**	**0.02**
Residence	Rural (ref)	--				--			
	Urban					1.04	0.61	1.78	0.88
Type of partner	Currently married/living with partner (ref)	--				--			
	Not married, no partner/boyfriend	**0.27**	**0.12**	**0.57**	**<0.01**	**0.07**	**0.01**	**0.42**	**<0.01**
Ever used FP	No (ref)	--				--			
	Yes	**2.01**	**1.07**	**3.77**	**0.03**	**2.27**	**1.41**	**3.66**	**<0.01**
Unmet need / Intention to use	Infecund/Menopausal/not sexually active & no intention to use (ref)	--				--			
	Infecund/Menopausal/not sexually active & intention to use >1 yr	1.09	0.37	3.22	0.88	1.55	0.43	5.56	0.50
	Infecund/Menopausal/not sexually active & intention to use <1 yr	2.31	0.51	10.48	0.27	3.82	0.65	22.51	0.14
	No unmet need & no intention to use	0.55	0.24	1.26	0.16	**2.90**	**1.26**	**6.71**	**0.01**
	No unmet need & intention to use > 1 yr	0.63	0.29	1.37	0.24	**4.36**	**1.55**	**12.30**	**0.01**
	No unmet need & intention to use ≤ 1 yr	**2.22**	**0.99**	**4.96**	**0.05**	**4.49**	**1.88**	**10.73**	**<0.01**
	Unmet need & no intention to use	2.38	0.87	6.52	0.09	2.78	0.98	7.86	0.05
	Unmet need & intention to use > 1 yr	1.86	0.74	4.71	0.18	**4.94**	**1.19**	**20.47**	**0.03**
	Unmet need & intention to use ≤ 1 yr	2.06	0.66	6.49	0.21	**8.95**	**3.03**	**26.41**	**<0.01**
N		665				2335			

		Rajasthan				Uganda			
		aOR	Lower CI	Upper CI	pvalue	aOR	Lower CI	Upper CI	pvalue
Age, years	15-24 (ref)	--				--			
	25-34	0.87	0.54	1.40	0.56	0.66	0.32	1.34	0.23
	35+	1.23	0.56	2.71	0.60	0.49	0.18	1.31	0.14
Highest Schooling level	None/Primary (ref)	--				--			
	Secondary+	1.72	0.93	3.20	0.08	1.58	0.90	2.77	0.11
Parity	0 children (ref)	--				--			
	1-2 children	**2.40**	**1.40**	**4.14**	**<0.01**	1.78	0.65	4.83	0.24
	3-4 children	**3.81**	**1.81**	**8.00**	**<0.01**	1.89	0.55	6.54	0.29
	5 plus children	2.33	0.90	6.03	0.08	2.47	0.77	7.90	0.12
Household wealth	Lower (ref)	--				--			
	Middle	0.82	0.39	1.70	0.58	1.29	0.69	2.40	0.40
	Highest	1.10	0.59	2.06	0.76	**1.46**	**1.04**	**2.07**	**0.03**
Residence	Rural (ref)	--				--			
	Urban	1.72	0.94	3.12	0.08	1.19	0.59	2.43	0.60
Type of partner	Currently married/living with partner (ref)	--				--			
	Not married, no partner/boyfriend	**0.14**	**0.05**	**0.41**	**<0.01**	0.54	0.24	1.22	0.13
Ever used FP	No (ref)	--				--			
	Yes	1.69	0.89	3.22	0.11	1.66	0.97	2.85	0.06
Unmet need / Intention to use	Infecund/Menopausal/not sexually active & no intention to use (ref)	--				--			
	Infecund/Menopausal/not sexually active & intention to use >1 yr	0.59	0.18	1.91	0.37	1.53	0.44	5.31	0.48
	Infecund/Menopausal/not sexually active & intention to use <1 yr	**3.66**	**1.33**	**10.09**	**0.01**	2.78	0.63	12.24	0.16
	No unmet need & no intention to use	**4.19**	**1.64**	**10.70**	**<0.01**	1.48	0.33	6.62	0.59
	No unmet need & intention to use > 1 yr	1.58	0.47	5.34	0.46	1.94	0.59	6.34	0.25
	No unmet need & intention to use ≤ 1 yr	**6.58**	**1.89**	**22.86**	**<0.01**	**3.26**	**1.08**	**9.83**	**0.04**
	Unmet need & no intention to use	2.04	0.69	6.00	0.19	0.99	0.24	4.13	0.99
	Unmet need & intention to use > 1 yr	**3.26**	**1.22**	**8.68**	**0.02**	2.73	0.58	12.76	0.19
	Unmet need & intention to use ≤ 1 yr	**7.56**	**2.45**	**23.33**	**<0.01**	**4.30**	**1.41**	**13.13**	**0.01**
N		2159				1922			
